# COVID-19-mediated patient delay caused increased total ischaemic time in ST-segment elevation myocardial infarction

**DOI:** 10.1007/s12471-021-01653-9

**Published:** 2022-01-19

**Authors:** H. N. Sturkenboom, V. A. E. van Hattem, W. Nieuwland, F. M. A. Paris, M. Magro, R. L. Anthonio, A. Algin, E. Lipsic, E. Bruwiere, B. J. L. Van den Branden, J. Polad, P. Tonino, R. A. Tio

**Affiliations:** 1 Department of Cardiology, Catharina Hospital, Eindhoven, The Netherlands; 2 Department of Cardiology, Antwerp University Hospital, Antwerp, Belgium; 3 Department of Cardiology, University Medical Centre Groningen, University of Groningen, Groningen, The Netherlands; 4 Department of Cardiology, Treant Zorggroep, Emmen, The Netherlands; 5 Department of Cardiology, Elisabeth-TweeSteden Hospital, Tilburg, The Netherlands; 6 Department of Cardiology, Amphia Hospital, Breda, The Netherlands; 7 Department of Cardiology, Jeroen Bosch Hospital, Den Bosch, The Netherlands; 8 Department of Educational Development and Research in the Faculty of Health, Medicine and Life Sciences, University of Maastricht, Maastricht, The Netherlands

**Keywords:** ST-segment myocardial infarction, COVID-19, Percutaneous coronary intervention, Treatment delay, Door-to-balloon time

## Abstract

**Background:**

The current study aimed to evaluate changes in treatment delay and outcome for ST-segment elevation myocardial infarction (STEMI) in the Netherlands during the first coronavirus disease 2019 (COVID-19) outbreak, thereby comparing regions with a high and low COVID-19 hospitalisation rate.

**Methods:**

Clinical characteristics, STEMI timing variables, 30-day all-cause mortality and cardiovascular complications of all consecutive patients admitted for STEMI from 1 January to 30 June in 2020 and 2019 to six hospitals performing a high volume of percutaneous coronary interventions were collected retrospectively using data from the Netherlands Heart Registry, hospital records and ambulance report forms. Patient delay, pre-hospital delay and door-to-balloon time before and after the outbreak of COVID-19 were compared to the equivalent periods in 2019.

**Results:**

A total of 2169 patients were included. During the outbreak median total treatment delay significantly increased (2 h 51 min vs 2 h 32 min; *p* = 0.043) due to an increased patient delay (1 h 20 min vs 1 h; *p* = 0.030) with more late presentations > 24 h (1.1% vs 0.3%) in 2020. This increase was particularly evident during the peak phase of COVID-19 in regions with a high COVID-19 hospitalisation rate. During the peak phase door-to-balloon time was shorter (38 min vs 43 min; *p* = 0.042) than in 2019. All-cause 30-day mortality was comparable in both time frames (7.8% vs 7.3%; *p* = 0.797).

**Conclusions:**

During the outbreak of COVID-19 patient delay caused an increase in total ischaemic time for STEMI, with a more pronounced delay in high-endemic regions, stressing the importance of good patient education during comparable crisis situations.

**Supplementary Information:**

The online version of this article (10.1007/s12471-021-01653-9) contains supplementary material, which is available to authorized users.

## What’s new?

- This study is the first to describe treatment delay in ST-segment elevation myocardial infarction (STEMI) pathways during the coronavirus disease 2019 (COVID-19) outbreak in the Netherlands.
- All STEMI timing variables, patient characteristics and outcome variables of 2169 patients presenting with STEMI to six high-volume percutaneous coronary intervention centres in the Netherlands during the COVID-19 outbreak and the equivalent period in 2019 were collected retrospectively.
- During the COVID-19 outbreak, total treatment delay significantly increased by 19 min due to an increased patient delay, particularly evident during the peak phase of COVID-19 in regions with high COVID-19 hospitalisation rates.
- Door-to-balloon time was significantly shorter in 2020 than in 2019.

## Introduction

The coronavirus disease 2019 (COVID-19) pandemic poses a major burden on health care systems worldwide and might negatively affect standard care for patients in need of urgent interventions. In the case of ST-segment elevation myocardial infarction (STEMI), percutaneous coronary intervention (PCI) reduces mortality when performed within guideline-recommended time frames, although the effect of the reduction of door-to-balloon time (DTB) to below 90 min and time from onset to door remains debatable [1–6]. Strains on emergency transportation facilities, hospital infrastructures and the capacity of catheterisation laboratories during the pandemic make timely revascularisation challenging. Despite STEMI-management algorithms, delays in seeking medical care for STEMI, increased treatment delay times as well as complication and mortality rates during the pandemic were reported [7–17].

To date, the impact of COVID-19 on STEMI pathways and related outcomes in the Netherlands remains unclear. Furthermore, the effect of regional hospitalisation rates for COVID-19 on treatment delay has not been described previously. We therefore performed a retrospective cohort study of all patients admitted for STEMI to six high-volume PCI centres in the Netherlands divided over regions with high and low hospitalisation rates for COVID-19. The objective was to quantify changes in treatment delay and related outcomes, comparing the pre-COVID-19 phase, the peak phase of COVID-19 in the Netherlands and the recovery phase in 2020 to the equivalent periods in 2019 and comparing high-endemic and low-endemic Dutch regions.

## Methods

### Study design and patient population

**Fig. 1:**
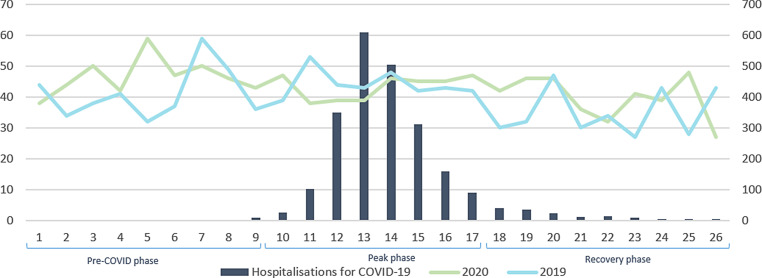
Weekly ST-segment elevation myocardial infarction (STEMI) presentations (*y‑axis, left*) at the six participating centres and the number of hospitalisations for COVID-19 per week (*y axis, right*) from 1 January to 30 June 2020 (*green line*) and the corresponding period in 2019 (*blue line*) with the number of hospitalisations for COVID-19 in 2020 per week divided into three phases: pre-COVID phase (January-February), peak phase of COVID-19 in the Netherlands (March-April) and recovery phase (May-June). Bar graphs show the number of hospitalisations for COVID-19 in the Netherlands per week

This multicentre retrospective observational cohort study included all patients aged 18 years or older with a discharge diagnosis of STEMI between 1 January and 30 June 2020 and the equivalent period in 2019 presenting to six participating high-volume PCI centres in the Netherlands (Electronic Supplementary Material, Table S1). Patients were selected based on ICD-10 codes (I21.0 to I12.3) collected by querying electronical medical record systems and this selection was cross-checked with local PCI datasets collected for the Netherlands Heart Registry (NHR). New hospitalisations for COVID-19 per week and per region at the peak of COVID-19 hospitalisations in week 13 were obtained from the National Institute for Public Health and the Environment (RIVM) [18–20]. Regions were labelled as either low-endemic or high-endemic according to colour-coded maps regarding regional differences in number of hospitalisations for COVID-19 per 100,000 inhabitants provided by the RIVM. Low-endemic was defined as 0‑9.6 hospitalisations for COVID-19 per 100,000 inhabitants and high-endemic as more than 9.6 hospitalisations [21] (Electronic Supplementary Material, Table S1). For analysis of the impact of COVID-19 on treatment delay, 2020 was subdivided into a pre-COVID-19 period (January-February) and a post-COVID-19 period with a peak phase (March-April) and recovery phase (May-June; Fig. 1; [19, 20]). The study was approved by the Medical Research Ethics Committees United (MEC‑U;2020.129).

### Study outcomes

Timing variables were extracted from catheterisation laboratory records. These data were complemented with data from ambulance report forms and hospital records. First medical contact (FMC) was defined as any contact with a medical professional regarding cardiac symptoms. In patients who presented through an emergency medical service (EMS), FMC was considered the time of the first call to the EMS. When the time of the first call to the EMS was missing, arrival of the ambulance at the patient’s location was used as the FMC. The following treatment delay times were ascertained: patient delay (onset of complaints—FMC), pre-hospital delay (FMC—arrival at PCI centre) and DTB (arrival—balloon inflation). After completion of data collection, system delay (diagnosis—arterial access) and total treatment delay (onset—arterial access) were added, given the availability of different timing variables in order to improve comparability between different times of interest.

NHR datasets were used for patient characteristics, cardiovascular risk factors, angiographic findings and secondary outcome variables. Missing data and data concerning clinical parameters, laboratory results, treatment type (PCI or no PCI), mechanical complications and repeat PCI ≤ 48 h were retrieved from hospital records.

Primary outcomes were changes in different types of treatment delay for STEMI during the outbreak of COVID-19 compared to the equivalent periods in 2019 and also between regions with high and low COVID-19 hospitalisation rates. All secondary outcomes are available in Appendix S1 (Electronic Supplementary Material).

### Statistical analysis

Baseline characteristics are mainly descriptive. Categorical variables are presented as absolute numbers with corresponding percentages. Continuous variables were tested for normality and are presented as mean (SD) or median [interquartile range (IQR)] as appropriate. Categorical variables were compared using a chi-square test or Fisher’s exact test where appropriate. Continuous variables were compared using an independent two-sample *t*-test for normally distributed variables and Mann-Whitney U test for skewed variables. A two-sided *p*-value of < 0.05 was considered significant for all analyses (SPSS Statistics, version 26.0; IBM Corp., Armonk, NY, USA).

## Results

### STEMI presentations

From January to June in 2019 and 2020 a total of 2169 patients were enrolled in this study. More STEMI presentations were observed during the COVID-19 year, 2020, mostly due to a higher rate before the first confirmed case in the Netherlands on 27 February. During the period after the COVID-19 outbreak (March-June 2020) 717 STEMI presentations were observed, compared to 684 in the equivalent period in 2019, reflecting a modest increase of 4.8% (Fig. 1).

### Patient characteristics and STEMI variables

Patient characteristics and angiographic findings in patients admitted between March and June 2020 and the equivalent period in 2019 are presented in Tab. 1 and the Electronic Supplementary Material (Table S2). The two groups did not differ in terms of age, gender distribution or history of coronary artery disease. Patients admitted for STEMI in 2020 more often had hypertension (49.8% vs 40.5%; *p* = 0.004) and hypercholesterolaemia (45.7% vs 37.7%; *p* = 0.016). In both time frames the majority of patients presented through an EMS (66.3% vs 70.1%). In 2020, 98.2% of patients underwent PCI versus 99.1% in 2019 (*p* = 0.130). In 2020, more presentations beyond 24 h after onset of complaints were registered (1.1% vs 0.3%) with higher cardiac troponin levels at admission (5.7 vs 4.6 times the 99th percentile of the upper reference limit; *p* = 0.029).

**Table 1:** Baseline characteristics and angiographic findings

	2020^a^	*N* = 717	2019^b^	*N* = 684	*p*-value
*Patient characteristics*					
Age (years)	64.07 ± 12.5	*n* = 717	63.7 ± 12.3	*n* = 684	0.490
Male	524 (73.1%)	*n* = 717	510 (74.6%)	*n* = 684	0.529
BMI (kg/m^2^)	26.3 (24.2–29.4)	*n* = 458	27.0 (24.3–29.6)	*n* = 430	0.262
Diabetes mellitus	108 (15.4%)	*n* = 700	106 (15.8%)	*n* = 669	0.832
Hypertension	245 (49.8%)	*n* = 492	181 (40.5%)	*n* = 447	0.004
Hypercholesterolaemia	209 (45.7%)	*n* = 457	159 (37.7%)	*n* = 422	0.016
Family history of CVD	198 (54.2%)	*n* = 365	175 (47.8%)	*n* = 366	0.082
Smoking	205 (44.1%)	*n* = 465	213 (47.9%)	*n* = 445	0.253
Prior myocardial infarction	89 (12.6%)	*n* = 709	89 (13.1%)	*n* = 681	0.773
Prior PCI	92 (12.9%)	*n* = 715	86 (12.6%)	*n* = 683	0.877
Prior CABG	24 (3.4%)	*n* = 716	25 (3.7%)	*n* = 684	0.758
Dialysis	4 (0.6%)	*n* = 717	1 (0.1%)	*n* = 684	0.375
cTn at admission	5.7 (1.9–27.4)	*n* = 370	4.6 (1.4–21.3)	*n* = 351	0.029
Maximum cTn	96 (34–271)	277	107 (33–248)	253	0.873
*Angiographic findings*					
Primary PCI performed^c^	704 (98.2%)	*n* = 717	678 (99.1%)	*n* = 684	0.130
No primary PCI performed	13 (1.8%)	*n* = 717	6 (0.9%)	*n* = 684	0.130
– Late presentation >12 h	1 (0.1%)		1 (0.1%)		
– Late presentation >24 h	8 (1.1%)		2 (0.3%)		
– Limited life expectancy	2 (0.3%)		2 (0.3%)		
– Unfavourable prognosis	2 (0.3%)		1 (0.1%)		

Values are mean ± SD, *n* (%), or median (IQR)

*BMI* body mass index, *CVD* cardiovascular disease, *PCI* percutaneous coronary intervention, *CABG* coronary artery bypass grafting, *cTn* cardiac troponin (number of times the 99th upper reference limit)

^a^2020 (COVID-19 year): 1 March to 30 June 2020

^b^1 March to 30 June 2019

^c^Every intervention with the intention of performing a primary PCI

### Treatment delay

**Fig. 2:**
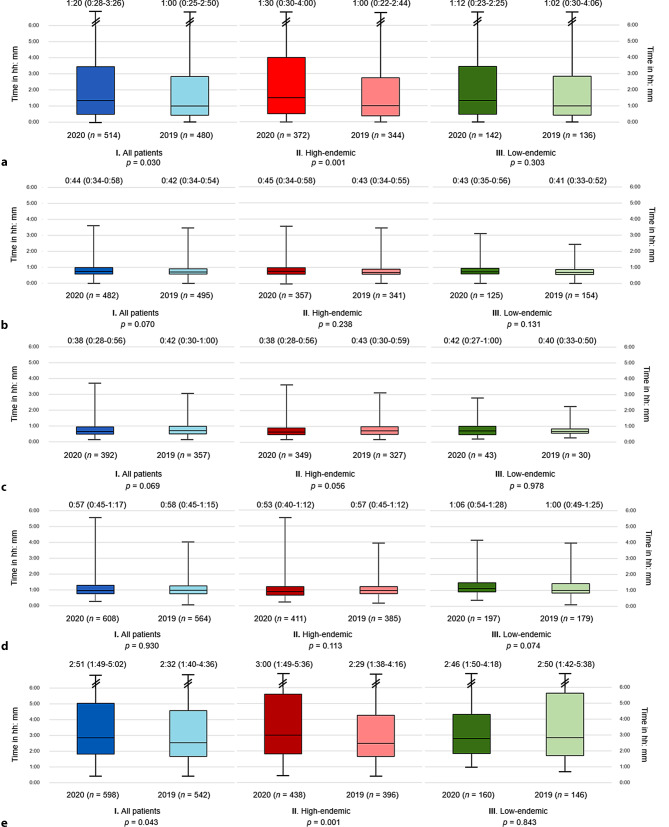
Box plot with different types of treatment delay in STEMI patients, comparing 2020 (COVID‑19 year; *n* = 717; 1 March to 30 June 2020) and 2019 (control period; *n* = 684; 1 March to 30 June 2019) and comparing highand low-endemic Dutch regions. **a** Patient delay = symptom onset—first medical contact. **b** Pre-hospital delay = first medical contact—arrival at PCI centre. **c** Door-to-balloon time = arrival at PCI centre—balloon inflation. **d** System delay = diagnosis—arterial access. **e** Total delay = symptom onset—arterial access. Values are median (IQR). *N* total number of patients. Delay times are in hours and minutes (hh:mm)

During the period after the COVID-19 outbreak in 2020 median total treatment delay times, expressed as hours and minutes (hh:mm), significantly increased from 2:32 (IQR: 1:40–4:36) in 2019 to 2:51 (IQR: 1:49–5:02) in 2020 (*p* = 0.043) (Fig. 2). Patient delay was the most important factor driving this delay [1:20 (IQR 0:28–3:26) in 2020 vs 1:00 (IQR 0:25–2:50) in 2019; *p* = 0.030]. Pre-hospital delay, system delay and DTB were comparable between time frames (Fig. 2).

When comparing the three different time phases in 2020 (Fig. 1) to the equivalent periods in 2019, patient delay [1:24 (IQR 0:27–3:52) vs 1:02 (IQR 0:30–2:20); *p* = 0.049] and pre-hospital delay [0:45 (IQR 0:35–0:59) vs 0:41 (IQR 0:33–0:52); *p* = 0.001] significantly increased during the peak phase. This resulted in an increase in median total treatment delay of 22 min [2:52 (IQR 1:51–5:40) vs 2:30 (IQR 1:40–4:07); *p* = 0.005] (Tab. 2). DTB significantly decreased by 5 min [0:38 (IQR 0:27–0:55) vs 0:43 (IQR 0:30–0:58); *p* = 0.042] during the peak phase. No significant differences were found when comparing treatment delay times in the pre-COVID-19 phase to those in the recovery phase (Tab. 2).

**Table 2:** Treatment delay in patients admitted for ST-segment elevation myocardial infarction during different phases in 2020 compared to the equivalent periods in 2019

Delay times (hh:mm)	2020^a^	*N* = 1131	2019^b^	*N* = 1038	*p*-value
*I. Pre-COVID phase*^c^	*Jan*–*Feb 2020*	*N* *=* *414*	*Jan*–*Feb 2019*	*N* *=* *354*	*p‑value*
Patient delay	1:13 (0:30–3:14)	*n* = 272	1:00 (0:30–2:49)	*n* = 239	0.354
Pre-hospital delay	0:44 (0:34–0:58)	*n* = 257	0:43 (0:33–0:55)	*n* = 251	0.308
DTB time	0:42 (0:32–1:00)	*n* = 221	0:42 (0:32–1:05)	*n* = 175	0.914
System delay	0:58 (0:43–1:17)	*n* = 341	0:55 (0:41–1:15)	*n* = 268	0.115
Total delay	2:57 (1:51–4:52)	*n* = 324	2:40 (1:44–5:10)	*n* = 264	0.256
*II. Peak phase*^d^	*Mar*–*Apr 2020*	*N* *=* *378*	*Mar*–*Apr 2019*	*N* *=* *378*	*p‑value*
Patient delay	1:24 (0:27–3:52)	*n* = 277	1:02 (0:30–2:20)	*n* = 259	0.049
Pre-hospital delay	0:45 (0:35–0:59)	*n* = 260	0:41 (0:33–0:52)	*n* = 266	0.001
DTB time	0:38 (0:27–0:55)	*n* = 207	0:43 (0:30–0:58)	*n* = 204	0.042
System delay	0:55 (0:45–1:18)	*n* = 320	0:57 (0:45–1:14)	*n* = 302	0.857
Total delay	2:52 (1:51–5:40)	*n* = 314	2:30 (1:40–4:07)	*n* = 295	0.005
*III. Recovery phase*^e^	*May*–*Jun 2020*	*N* *=* *339*	*May*–*Jun 2019*	*N* *=* *306*	*p‑value*
Patient delay	1:13 (0:29–3:07)	*n* = 237	1:00 (0:20–3:43)	*n* = 221	0.259
Pre-hospital delay	0:44 (0:34–0:57)	*n* = 222	0:45 (0:35–0:56)	*n* = 229	0.439
DTB time	0:40 (0:31–1:00)	*n* = 185	0:42 (0:29–1:01)	*n* = 153	0.610
System delay	0:58 (0:45–1:16)	*n* = 288	1:00 (0:48–1:16)	*n* = 262	0.622
Total delay	2:51 (1:45–4:35)	*n* = 284	2:35 (1:39–5:09)	*n* = 247	0.354

*Patient delay* symptom onset—first medical contact, *Pre-hospital delay* first medical contact—arrival at PCI centre, *System dela*y diagnosis—arterial access, *DTB time* door-to-balloon time = arrival PCI centre—balloon inflation, *Total delay* symptom onset—arterial access. Values are median (IQR). *N* total number of patients. Delay times are in hours and minutes (hh:mm)

^a^2020 (COVID-19 year; *n* = 1131): 1 Januaryto 30 June 2020

^b^2019 (control period; *n* = 1038): 1 January to 30 June 2019

^c^Pre-COVID-19 period (January–February 2020; *n* = 414)

^d^Peak phase (March–April 2020; *n* = 378)

^e^Recovery phase (May–June 2020; *n* = 339)

When comparing high-endemic and low-endemic regions, total treatment delay significantly increased only in high-endemic regions [3:00 (IQR 1:49–5:36) vs 2:29 (IQR 1:38–4:16); *p* = 0.001], which was mostly explained by an increase in patient delay [1:30 (IQR 0:30–4:00) vs 1:00 (IQR 0:22–2:44); *p* = 0.001]. No significant differences in treatment delays were found in low-endemic regions (Fig. 2).

### Patient outcome: complication and mortality rate

Despite the increase in treatment delay during the peak phase, the observed all-cause 30-day mortality was comparable to that in 2019 (7.8% vs 7.3%; *p* = 0.797). Secondary outcomes did not differ significantly (Tab. 3).

**Table 3:** Patient outcome: complications and all-cause mortality of ST-segment elevation myocardial infarction patients during the peak phase of COVID-19 in the Netherlands (1 March to 30 April 2020) compared to the control group (1 March to 30 April 2019)

Outcome variable	2020	*N* = 378	2019	*N* = 378	*p*-value
OHCA	39 (10.3%)	*n* = 378	39 (10.3%)	*n* = 378	1.000
Cardiogenic shock	29 (7.7%)	*n* = 375	34 (9.0%)	*n* = 378	0.532
Mechanical complication	1 (0.4%)	*n* = 241	3 (1.3%)	*n* = 234	0.431
– VSR	0 (0.0%)		0 (0.0%)		
– FWR	0 (0.0%)		1 (0.4%)		
– IMR	1 (0.4%)		2 (0.9%)		
Urgent CABG ≤ 24 h	2 (0.5%)	*n* = 378	4 (1.1%)	*n* = 378	0.686
Repeat PCI ≤ 48 h	4 (1.1%)	*n* = 378	6 (1.6%)	*n* = 378	0.542
Myocardial re-infarction ≤ 30 days	3 (1.1%)	*n* = 284	6 (1.7%)	*n* = 360	0.738
All-cause mortality ≤ 30 days	29 (7.8%)	*n* = 372	27 (7.3%)	*n* = 370	0.797

*OHCA* out of hospital cardiac arrest, *VSR* ventricular septal rupture, *FWR* free wall rupture, *IMR* ischaemic mitral regurgitation, *CABG* coronary artery bypass grafting, *PCI* percutaneous coronary intervention

## Discussion

In the present study, a significant increase in total treatment delay of 19 min was observed during the COVID-19 outbreak, with patient delay being the most important driving factor. Patient delay more specifically increased during the peak phase and was more pronounced in high-endemic regions. During the pandemic, patient delay has previously been described with avoidance of medical care due to lockdown measures, a general fear of contracting COVID-19, confusion of cardiac complaints with COVID-19-related symptoms and restraint from burdening the hospitals suggested to be responsible for these delays [9–11]. This effect might even be more evident in high-endemic regions when compared to low-endemic regions, although patient delay has previously been reported in regions with a low incidence rate of COVID-19 [9]. Although the longer delay between onset of complaints and FMC is most likely patient-related, we cannot exclude an additional effect on delay times of more restricted access to health care facilities. During the peak phase a significant increase in pre-hospital delay of 4 min was observed, which may also reflect a health care provider’s delay, for instance by confusing STEMI complaints with COVID-like symptoms or limited resources. Given the retrospective nature of our study no causal relations could be established.

Interestingly, DTB significantly decreased by 5 min during the peak phase of COVID-19, suggesting that despite restructuring of health care systems in-hospital STEMI pathways were highly efficient. In the European position statement on myocardial infarction during the COVID-19 pandemic, a delay in PCI pathways up to 60 min was anticipated [7]. In Belgium, median DTB was prolonged by 6 min, while a British national study reported an additional DTB of 11 min [10, 12]. When comparing hospitalisation rates for COVID-19 per million inhabitants, the United Kingdom was more severely affected than Belgium and the Netherlands and thus might have suffered an additional burden on health care systems, leading to a more pronounced delay [24]. On the other hand, although Ohio was regarded as a non-COVID-19 epicentre, DTB in Ohio was significantly longer during the pandemic (35 min) [9]. The cancellation of most elective procedures together with the high density of PCI-capable hospitals and efficient in-hospital regulatory changes of STEMI pathways most likely were responsible for the decrease in DTB in the Netherlands during the outbreak of COVID-19.

During the peak phase, total ischaemic time was significantly prolonged with a higher cardiac troponin level at admission. Although this imputes more severe cardiac damage, it did not adversely influence short-term adverse events or 30-day mortality. This is in agreement with findings in England, where a significant increase in both symptom-to-door time and DTB did not negatively affect outcome [10]. The absence of a significant change might be the result of a DTB remaining persistently below the recommended 90 min in 93% of cases and the effect of reduction of DTB below these time targets remains debatable [4, 6]. Conversely, since patient delay was the driving factor, it might also reflect the fact that more patients died before arrival at a PCI-capable hospital, resulting in a survivor-cohort effect, whereby those who present to the hospital have already survived the period with the highest risk of death [5]. The true effect of a pre-hospital delay on mortality therefore remains difficult to ascertain.

Our study further shows a modest increase in STEMI admissions of 4.8% in the period after the COVID-19 outbreak when compared to 2019. Although reductions of up to 43% were reported worldwide, admission rates for STEMI in New Zealand and Germany were comparable to those in the previous year and no causal relation between hospitalisation rate for COVID-19 and STEMI presentations could be established previously [8, 9, 12, 14, 17, 22–24]. The reduction in STEMI admissions in general might be the result of patients’ postponements of hospital visits or STEMI cases being missed for a variety of reasons, and this effect might differ between nations. The absence of an evident reduction in admissions in this study may partially be the result of our study design. Patients were selected based on ICD-10 codes, meaning patients who did not undergo PCI were also included, while these patients were excluded from other registries using catheterisation laboratory procedures as the inclusion method. However, since the increase in the number of patients who did not undergo PCI for STEMI was limited (1.8% in 2020 vs 0.9% in 2019), this might only explain the small percentage differences. Nationwide a reduction up to 34% in week 14 was reported, using data from the National Basic Hospital Care Registry (LBZ), in which patients with a myocardial infarction within the 2 years preceding the event were excluded [25]. Since our study specifically focused on treatment delay, using high-quality data with regard to timing variables in the extremes of high-endemic and low-endemic regions in the Netherlands, our study design may not be suitable to make a statement on the number of STEMI presentations on a national level. Furthermore, previously published reports regarding STEMI presentations during the COVID-19 pandemic showed large methodological differences regarding inclusion methods and quality of data collection. Moreover, in some cases data were collected on a regional level and in others on a national level; hence, true comparisons between countries may be difficult.

The aforementioned results should be interpreted in the light of the following limitations. First, because of the observational nature of this study, no causal relation could be established. Second, since the data were collected retrospectively, not all data were available for analysis. Registration of STEMI timing variables in catheterisation laboratory forms varied between hospitals and although timing variables were supplemented with ambulance report forms and hospital records, not all timing variables could be retrieved.

In conclusion, although STEMI pathways for primary PCI were highly efficient during the COVID-19 pandemic, increased patient delay is a serious concern causing an increase in total ischaemic time, stressing the importance of timely education of patients during comparable crisis situations. While our study did not demonstrate an increase in short-term adverse events and 30-day mortality, extra awareness regarding possible long-term consequences is warranted.

## Supplementary Information

Appendix S1: Additional secondary outcomes
Table S1: Participating hospitals and geographic location with hospital admissions for COVID-19 per 100 000 inhabitants in week 13
Table S2: Baseline characteristics and angiographic findings
