# Supplementary material for: COVID-19-mediated patient delay caused increased total ischaemic time in ST-segment elevation myocardial infarction
Source: Neth Heart J. 2022 Jan 19;30(2):96–105. doi: 10.1007/s12471-021-01653-9 (PMC8767528; doi:10.1007/s12471-021-01653-9)
Supplement: Supplementary file 1 — Appendix S1: Additional secondary outcomes Table S1: Participating hospitals and geographic location with hospital admissions for COVID-19 per 100 000 inhabitants in week 13 Table S2: Baseline characteristics and angiographic findings [file 12471_2021_1653_MOESM1_ESM.docx]

**Electronic Supplementary material:**

**Table S1:** Participating hospitals and geographic location

| Participating hospital | City | Hospital admissions per 100 000 inhabitants in week 13 | High/low endemic^*^ |
| --- | --- | --- | --- |
| Jeroen Bosch Hospital | ‘s Hertogenbosch | 29.0 | High |
| Amphia Hospital | Breda | 19.6 | High |
| Elisabeth-TweeSteden Hospital | Tilburg | 35.9 | High |
| Catharina Hospital Eindhoven | Eindhoven | 31.1 | High |
| University Medical Centre Groningen | Groningen | 3.4 | Low |
| Treant Zorggroep Emmen | Emmen | 9.3 | Low |

**Table S1:** Participating hospitals and geographic location with hospital admissions per 100 000 inhabitants in week 13 according to the National Institute for Public Health and the environment^[21]^ ^*^ High-endemic region: Dutch region with more than 9.6 new hospitalisations for COVID-19 per 100 000 inhabitants in week 13. Low-endemic region: Dutch region with 0-9.6 new hospitalisations for COVID-19 per 100 000 inhabitants.

**Table S2:** baseline characteristics and angiographic findings

|  | 2020* | | N =717 | 2019† | N =684 | | | p-value |
| --- | --- | --- | --- | --- | --- | --- | --- | --- |
| *Referral route* |  | |  |  |  | | |  |
| FMC type |  | | n = 707 |  | n = 673 | | | 0,316 |
| - EMS | 469 (66.3%) | |  | 472 (70.1%) |  | | |  |
| - General practitioner | 155 (21.9%) | |  | 141 (21 %) |  | | |  |
| - ED referring hospital | 53 (7.5%) | |  | 38 (5.6%) |  | | |  |
| - ED PCI performing hospital | 30 (4.2%) | |  | 22 (3.3%) |  | | |  |
| *Clinical parameters* |  | |  |  |  | | |  |
| SBP (mmHg) | 132 ± 27 | | n = 477 | 131 ±28 | n = 452 | | | 0,604 |
| DBP (mmHg) | 77 ± 17 | | n = 477 | 76 ± 17 | n = 452 | | | 0,530 |
| Heart rate (beats/min) | 74 (63-86) | | n = 475 | 74 (63-85) | n = 455 | | | 0,599 |
| Oxygen saturation (%) | 97 (95-98) | | n = 449 | 97 (95-98) | n = 423 | | | 0,164 |
| Respiratory rate (/min) | 16 (14-19) | | n = 400 | 17 (15-19) | n = 331 | | 0,240 | |
| Serum creatinine (µmol/l) | 79 (67-92) | | n = 634 | 80 (69-94) | n = 641 | | | 0,195 |
| *Angiographic findings* | |  |  |  | |  | |  |
| Arterial access | |  | N=704 |  | |  | |  |
| - Radialis | | 571 (81,1%) |  | 562 (82,9%) | | N=678 | | 0,700 |
| - Femoralis | | 132 (18,8%) |  | 115 (17%) | |  | |  |
| - Brachialis | | 1 (0,1%) |  | 1 (0,1%) | |  | |  |
| Culprit vessel | |  | n = 704 |  | | n = 678 | | 0,531 |
| - No culprit found | | 19 (2.7%) |  | 22 (3.2%) | |  | |  |
| - LM | | 15 (2.1%) |  | 10 (1.5%) | |  | |  |
| - LAD | | 251 (35.7%) |  | 272 (40.1%) | |  | |  |
| - RCX | | 98 (13.9%) |  | 102 (15%) | |  | |  |
| - AL/IM | | 7 (1.0%) |  | 5 (0.7%) | |  | |  |
| - RCA | | 305 (43.3%) |  | 259 (38.2%) | |  | |  |
| - Venous graft | | 7 (1.0%) |  | 7 (1.0%) | |  | |  |
| - Arterial graft | | 2 (0.3%) |  | 1 (0.1%) | |  | |  |
| Multivessel disease | | 358 (50.9%) | n = 704 | 316 (46.6%) | | n = 678 | | 0,115 |
| CTO | | 11 (1.6%) | 2=704 | 11 (1.6%) | | n = 678 | | 0,929 |

**Table S2:** Values are mean ± SD, n (%), or median (IQR). *2020 (COVID-19 year): 01/03/2020-30/06/2020. †2019 (control period): 01/03/2019-30/06/2019; FMC: First medical contact; EMS = Emergency Medical Services; ED = Emergency Department; SBP = systolic blood pressure; DBP = diastolic blood pressure; LM = left main; LAD = left anterior descending; RCX = ramus circumflexus; AL/IM = anterolateral/intermediate branch; RCA = right coronary artery; CTO = chronic total occlusion.

**Appendix S1:** Secondary outcomes

Secondary outcomes were differences in outcome variables: out of hospital cardiac arrest (OHCA), mechanical complications (ventricular septal rupture, free wall rupture, ischemic mitral regurgitation), cardiogenic shock, urgent CABG ≤24 hours, re-PCI ≤24 hours, myocardial re-infarction ≤30 days, mortality ≤30 days.
